# How Management and Leadership Training Can Impact a Health System: Evaluation Findings From a Public Health Management Training Program in Cambodia

**DOI:** 10.3389/fpubh.2021.784198

**Published:** 2022-01-26

**Authors:** Chelsea Horváth, Kimsear Hong, Paulah Wheeler, Por Ir, Chhorvann Chhea, Michael H. Kinzer, Vanthy Ly, Erika Willacy

**Affiliations:** ^1^Consultant to Training Programs in Epidemiology and Public Health Interventions Network, Boldogasszonyfa, Hungary; ^2^National Institute of Public Health, Phnom Penh, Cambodia; ^3^Division of Global HIV and TB, Center for Global Health, U.S. Centers for Disease Control and Prevention, Pretoria, South Africa; ^4^Division of Global Health Protection, Center for Global Health, U.S. Centers for Disease Control and Prevention, Atlanta, GA, United States; ^5^Division of Global Health Protection, Center for Global Health, U.S. Centers for Disease Control and Prevention, Phnom Penh, Cambodia; ^6^Division of Global Health Protection, Center for Global Health, U.S. Centers for Disease Control and Prevention, Boston, MA, United States

**Keywords:** management, leadership, health system, evaluation, Cambodia, health workforce capacity building, training

## Abstract

In 2017, the National Institute of Public Health in Cambodia collaborated with the U.S. Centers for Disease Control and Prevention to provide management and leadership training for 20 managers and senior staff from 10 health centers. We conducted a mixed methods evaluation of the program's outcomes and impact on the graduates and health centers. From June 2018 (baseline) to January 2019 (endpoint), we collected data from a competency assessment, observational visits, and interviews. From baseline to endpoint, all 20 participants reported increased competence in seven management areas. Comparing baseline and endpoint observational visits, we found improvements in leadership and governance, health workforce, water, sanitation, and hygiene, and health centers' use of medical products and technologies. When evaluating the improvements made by participants against the World Health Organization's key components of a well-functioning health system, the program positively contributed toward building four of the six components—leadership and governance, health information systems, human resources for health, and service delivery. While these findings are specific to the context of Cambodian health centers, we hope this evaluation adds to the growing body of research around the impact of skilled public health management on health systems.

## Introduction

As a complex adaptive system, health care and health systems demand competent managers and transformational leaders throughout its organizations to optimize health outcomes. Frontline health managers are often overlooked in health systems strengthening research and practice, although they directly influence the health care workers and the operational levels of the health care system responsible for delivering products and services to the population (1).

In an effort to improve coverage and the quality of health care services, the Government of Cambodia introduced health care reform in 1995, classifying the public health system into national and provincial levels. In each province, operational districts were formed based on the size of the population. Referral hospitals provide level one to level three specialist services depending upon the area. Health centers play an integral role in the Cambodian health system, providing frontline health services to the public; each health center provides basic primary care services to populations of 8,000 to 10,000 persons. Health centers receive feedback and overall guidance from the community through a health center management committee (HCMC) and are under the management and supervision of the operational district (2). A health center chief leads the daily activities of the health center, generally supervising a deputy chief and at least seven additional staff members. The health center chief also operationalizes health policy from national and sub-national levels to support health center staff and the communities they serve.

Despite their integral role, health center chiefs do not receive management and leadership training prior to assuming their positions. Thus, in 2017 the National Institute of Public Health (NIPH) in Cambodia collaborated with the U.S. Centers for Disease Control and Prevention (CDC) to address this need and provide management and leadership training for its health center managers and senior staff. With the approval of the Cambodia Ministry of Health, NIPH formed a technical working group to discuss the necessary knowledge and skills a health center manager needs. The technical working group included representatives from the national and sub-national offices of the Cambodia Ministry of Health, NIPH, CDC, and the Korea International Cooperation Agency (KOICA). After a series of workshops, the technical working group developed a 6-month management and leadership capacity building program, utilizing technical support from CDC and financial support from KOICA. The program was adapted from the Improving Public Health Management for Action (IMPACT) program, originally launched by the CDC in 2016 (3).

Previous thought scholarship in global health workforce development has acknowledged that better management is a necessary component to improving global health outcomes. Bradley et al. argued that a three-part strategy is needed to build the field of management as a key pillar of global health: training and education, practice, and research (4). Additionally, Yeager and Bertrand note that little evidence exists to inform public health management needs and best practices in global health settings (5–7). Thus, this paper will assess evaluation findings from the IMPACT Cambodia program and evaluate these findings against the World Health Organization's (WHO) key components of a well-functioning health system (Table 1). While these findings are specific to the context of Cambodian health centers, we hope this evaluation adds to the growing body of research around the impact of skilled public health management on health systems. In sharing the findings of our evaluation, we aim to contribute to the discussion around the importance of public health management in health systems strengthening.

**Table 1:** IMPACT Cambodia classroom modules and the related World Health Organization's key components of a well-functioning health system (8).

**IMPACT Cambodia classroom modules**	**Related WHO key components of a well-functioning health system**
Organizational leadership and systems awareness	Leadership and governance
Community and health center engagement and communication	Health information systems
Achieving quality of care through supportive supervision, delegation, and teamwork	Human resources for health; service delivery
Planning and operational management	Leadership and governance
Budgeting and financial management for successful outcomes: developing standard operating procedures	Leadership and governance; service delivery; health financing
Emergency planning, preparedness, and response	Leadership and governance

*IMPACT, Improving Public Health Management for Action; WHO, World Health Organization*.

## Materials and Methods

The NIPH Technical Working Group selected Battambang and Preah Vihear provinces in northern Cambodia to pilot the IMPACT Cambodia program. Participants were nominated by their Provincial Health Directors and selected by NIPH. IMPACT Cambodia aimed to build capacity through interactive classroom training and regular mentorship at health center sites. Classwork was comprised of 6, 1-week modules covering topics listed in Table 1. The last module of the program was a symposium, providing participants with the opportunity to share how they have implemented lessons learned. After the first five training modules, a team of mentors visited each health center to provide technical guidance on fellows' activities, answer lingering questions from the modules, and provide direct coaching to the fellows. NIPH recruited seven individuals from its staff to serve on the mentorship team; mentors were not involved in the technical working group or other aspects of program implementation. However, mentors were provided with mentorship training by CDC prior to program initiation in August 2018.

The evaluation of the IMPACT Cambodia program utilized a mixed methods approach, employing a baseline and endpoint competency assessment, observational visits, and interviews. The technical working group provided direction and technical support for program implementation and evaluation design. The evaluation's goal was to determine the extent to which the program reached its intended outcome (participants' increased management competence) and what impact the program had on graduates and health centers.

The evaluation received a non-research determination by the CDC Center for Global Health's Institutional Review Board (Tracking number: 2018-169) and approved by the Cambodia National Ethics Committee for Health Research (#134NECHR; 21 June 2018). The key ethical principles of voluntary and informed participation, confidentiality and safety of participants was used in evaluator and participant interactions. Participants were provided with written information about the evaluation, informed that their participation was voluntary, and that they may withdraw from participation at any time.

From June 2018 to January 2019, data were collected from the 20 participants (nine health center chiefs and 11 health center staff members) from 10 health centers in Battambang and Preah Vihear provinces in Cambodia. Quantitative methods were used to measure the change in participant competence and at the health centers; qualitative methods were deployed to provide context to those changes. Analytic approaches varied by data source and type. Data sources and respective analytic approaches included the following:

### Competency Assessment

To measure the competency change for participants, we utilized baseline (August 2018) and endpoint (January 2019) competency assessments. We asked participants to rank their knowledge and skills in performing selected tasks in seven management areas: analytics and assessment; communications; program planning; community engagement; financial planning and management; leadership and systems thinking; and emergency preparedness, planning and response. Participants ranked their knowledge and skills in each area on a Likert scale of one to four as described in Table 2. The assessment was created by the Council of Linkages (9) which was validated by Edgar et al. in their study of the construct validity and reliability of the Council of Linkages' core competencies for public health professionals (10). The assessment and was translated into Khmer, Cambodia's official language. To ensure accurate translation, we used human translation with an additional review by our research assistant, a native Khmer speaker. Using R statistical software, we calculated the mean and standard deviation for each management competency at the baseline and endpoint (11). We conducted paired *t*-tests to compare baseline and endpoint for each management competency and set statistical significance at *p* < 0.01.

**Table 2:** Likert scale for baseline and endpoint competency assessment, created by the Council of Linkages (9).

1 = None; I am unaware or have very little knowledge of the skill.	3 = Knowledgeable; I am comfortable with my knowledge or ability to apply the skill.
2 = Aware; I have heard of but have limited knowledge or ability to apply the skill.	4 = Proficient; I am very comfortable, am an expert, or could teach this skill to others.

### Observational Visit Quantitative Checklist

At baseline (June/July 2018) and endpoint (January 2019), we visited all 10 health centers to conduct observational visits. To reduce observer bias, one to three members of the evaluation team visited each health center with the aim of yielding more comprehensive, accurate observational data (12). In health centers where more than one observer was present, this also helped to provide inter-observer reliability in completion of the checklist.

We completed a quantitative checklist of indicators related to the management of each health center. The checklist used closed-ended questions and unambiguous response categories to reduce observer bias. The checklist was unique to the context of health centers in Cambodia; the evaluation team and key technical working group members developed the checklist based on the curriculum, and roles and responsibilities of health center chiefs and staff. It covered seven categories (service delivery; health information systems; leadership and governance; health workforce; water, sanitation, and hygiene [WASH]; health financing; and medical products and technology), with a total of 41 indicators (Table 3). The evaluation team scored indicators through direct observation or document review. Observations were recorded promptly to reduce bias. Checklist results were compiled in Microsoft Excel. Nominal values were averaged, and frequencies calculated, where necessary.

**Table 3:** Change in indicators, from baseline to endpoint, of IMPACT Cambodia participants' health centers, by health system category.

**Category and Indicator**	**Percentage of Health Centers Where Observed**		**Average Number**	**Average Number**	**Change, If Applicable**
	**Baseline**	**Endpoint**	**Baseline**	**Endpoint**	
Service Delivery				
Average population that the health center covers (number)	N/A	N/A	11,656	N/A	No change
Average volume of patients, monthly (number)	N/A	N/A	780	N/A	No change
Positive attitude when speaking with patients (observe)	100%	100%	N/A	N/A	No change
Floors are clean (observe)	80%	80%	N/A	N/A	No change
Surfaces of beds and tools are sanitized after patient (observe)	100%	100%	N/A	N/A	No change
Health center documents are organized (observe)	100%	100%	N/A	N/A	No change
Existence of ongoing health education campaign materials (Pamphlets, posters, flyers, brochures)	100%	100%	N/A	N/A	No change
Discuss health education campaign with patients	100%	100%	N/A	N/A	No change
Health Information System					
Patient record log	100%	100%	N/A	N/A	No change
White board used to display health center indicators (observe)	100%	100%	N/A	N/A	No change
White board updated monthly with health center indicators (observe)	100%	100%	N/A	N/A	No change
Percentage of HC1 forms reviewed complete (observe)	100%	100%	N/A	N/A	No change
Leadership and Governance					
Average # of days since last all-staff meeting	N/A	N/A	32	35	+3 days
Minutes from last all-staff meeting	100%	100%	N/A	N/A	No change
Average # of days since last health center committee (HCMC) meeting	N/A	N/A	96	67	−29 days
Minutes from last HCMC meeting	100%	100%	N/A	N/A	No change
Existence of monthly or quarterly plan for the health center	60%	90%	N/A	N/A	+30%
Existence of annual plan for the health center	100%	100%	N/A	N/A	No change
Existence of data used when developing the monthly or quarterly plans for the health center	60%	90%	N/A	N/A	+30%
Existence of data used when developing the annual plans for the health center	100%	100%	N/A	N/A	No change
Existence of outbreak/disaster preparedness plan	10%	40%	N/A	N/A	+30%
Existence of outbreak/disaster plan	10%	40%	N/A	N/A	+30%
Existence of post-outbreak/disaster plan	10%	0%	N/A	N/A	−10%
Existence of plan to communicate with community about outbreak/disaster	0%	0%	N/A	N/A	No change
Plan to mobilize community resources for disaster response	0%	0%	N/A	N/A	No change
Health Workforce					
Evidence of minimum required education for all staff roles (refer to Minimum Packet Activity)	10%	100%	N/A	N/A	+90%
Staff duty roster filled	90%	100%	N/A	N/A	+10%
Staff absenteeism or tardiness tracked	100%	100%	N/A	N/A	No change
Formal performance reviews conducted	50%	80%	N/A	N/A	+30%
Water, Sanitation, and Hygiene					
Water from an improved source is available on premises	100%	100%	N/A	N/A	No change
Separate toilets available for patients and staff	30%	50%	N/A	N/A	+20%
Separate toilets are available for men and women, including allowing menstrual hygiene management	90%	100%	N/A	N/A	+10%
Existence of toilets meeting the needs of handicapped people	20%	50%	N/A	N/A	+30%
Hand hygiene materials, either a basin with water and soap or alcohol hand rub, are available at points of care	100%	90%	N/A	N/A	−10%
Hand hygiene materials, either a basin with water and soap or alcohol hand rub, are available at toilets	100%	90%	N/A	N/A	−10%
There are three waste bins in consultation room: one for normal waste, one for sharp waste (i.e., needles), and one for infectious waste	100%	100%	N/A	N/A	No change
Health Financing					
Existence of complete annual budget for money from national government	100%	100%	N/A	N/A	No change
Existence of complete annual budget for direct service grant money	100%	100%	N/A	N/A	No change
Existence of document comparing actuals with budget plan	70%	70%	N/A	N/A	No change
Medical Products & Technology					
Existence of updated supply list of drugs (observe)	100%	100%	N/A	N/A	No change
Drugs reviewed are current/not expired (observe)	70%	100%	N/A	N/A	30%

*IMPACT, Improving Public Health Management for Action; N/A, not applicable; HC1, health centers' monthly reports; HCMC, health center committee meeting*.

### Interviews

In addition to the checklist, we conducted semi-structured interviews with all 20 participants from each health center at baseline and 19 participants at endpoint. We were unable to interview one participant at endpoint due to illness. Participants were asked about management processes and challenges at baseline and endpoint, as well as if and how they are applying skills learned from the training (Table 4). All interviews were conducted in Khmer by a trained evaluator. Interview data were translated by a native Khmer speaker to English for analysis.

**Table 4:** Questions asked during semi-structured interviews with IMPACT Cambodia participants, at baseline and endpoint.

**Baseline interview questions**	**Endpoint interview questions**
What is your professional background? How long have you been in this position? What are your future career plans?	Tell us about your overall experience with the IMPACT program. What were your expectations of the program and did it meet your expectations? What are your future career plans?
What are the current health issues or challenges in the local community?	What are the current health issues or challenges in the local community? Has the IMPACT program helped you with this? [If yes, what specific IMPACT courses or topics helped you?]
What are the current management challenges in the health center?	What are the current management challenges in the health center? Has the IMPACT program helped you with this? [If yes, what specific IMPACT courses or topics helped you?]
How do you implement the monthly or quarterly plan?	How do you plan to implement the annual, monthly or quarterly plan? Has the IMPACT program helped you with this? [If yes, what specific IMPACT courses or topics helped you?]
Do you communicate with the media about outbreaks and health issues in the community? If yes, how? If no, please explain why not?	Do you plan to communicate with the media about outbreaks and health issues in the community? If yes, how? If no, please explain why not?
Do you have rules or procedures to manage conflict among health center staff? If yes or no, please explain briefly	Do you have rules or procedures to manage conflict among health center staff? If yes or no, please explain briefly. Has the IMPACT program helped you with this? [If yes, what specific IMPACT courses or topics helped you?]
Do you meet with staff to discuss professional development opportunities? If yes or no, please explain briefly?	Do you meet with staff to discuss professional development opportunities? If yes or no, please explain briefly? Has the IMPACT program helped you with this? [If yes, what specific IMPACT courses or topics helped you?]
What is the process to prepare the health center budgets?	How do you plan to prepare the health center budget? [If you are not in charge of the budget, how do you plan to help with the preparation?] Has the IMPACT program helped you with this? [If yes, what specific IMPACT courses or topics helped you?]
How do you collect input from the community?	How do you plan to collect input from the community? Has the IMPACT program helped you with this? [If yes, what specific IMPACT courses or topics helped you?]
	Many final presentations discussed the SOPs that they created as a result of the training. What do you plan to do with these SOPs? What is the next step? [Do you plan to train your staff on the SOPs? If so, when?]
	Tell us about your mentorship experience. [Did it meet your expectations at the beginning of the program? Why or why not? Was mentorship useful for you in terms of technical skills, goal setting, and professional opportunities?]
	Tell us about your experience with the New Generation of Leadership module and/or the Liberating Structures power hours? Has it made an impact in your work at the health center? If yes, please explain how. If no, please explain why it was not helpful.
	Do you have any suggestions for improvements to the program?

*IMPACT, Improving Public Health Management for Action; SOPs, Standard Operating Procedures*.

We conducted an analytic induction of the qualitative data using Microsoft Excel and ATLAS.ti. We started the analysis with deductive themes based on the evaluation goals and continued to look for undiscovered patterns and emergent themes throughout the analysis (13). We used this qualitative analysis method to ensure that we were meeting the evaluation goals, while also allowing for unexpected themes to emerge.

## Findings

### Baseline and Endpoint Competency Assessment

Participants reported increased competence in all management areas (Table 5). The response rates for both baseline and endpoint competency assessments were 100%. All *t*-values were negative and statistically significant (*p* < 0.01).

**Table 5:** Change in mean competence, from baseline to endpoint, of IMPACT Cambodia participants, by management area.

	**Baseline**		**Endpoint**		**Change Baseline to Endpoint**	
**Management Area**	**Mean (SD)**		**Mean (SD)**		**Mean**	**(SD)**
Emergency planning, preparedness and response	1.43	(0.54)	2.57	(0.45)	+1.14	(0.58)
Leadership and systems thinking	1.58	(0.53)	2.68	(0.36)	+1.10	(0.43)
Program planning	1.48	(0.44)	2.57	(0.32)	+1.09	(0.39)
Financial planning and management	1.52	(0.48)	2.50	(0.46)	+0.98	(0.50)
Analysis and assessment	1.59	(0.39)	2.55	(0.33)	+0.96	(0.36)
Communications	1.68	(0.43)	2.64	(0.30)	+0.96	(0.37)
Community engagement	1.65	(0.47)	2.54	(0.40)	+0.89	(0.49)

*IMPACT, Improving Public Health Management for Action; SD, standard deviation. Competence units defined in Table 2*.

### Observational Visit Quantitative Checklist

Comparing baseline and endpoint observational visits, we found improvements in four of the seven categories: leadership and governance, health workforce, WASH, and medical products and technology (Table 3). For leadership and governance, we found increases in the use of monthly and quarterly plans; the use of data when developing the monthly and quarterly plans; the development of outbreak/disaster preparedness plans; and the creation of outbreak/disaster plans.

Findings indicated that the average number of days since the last health center committee (HCMC) meeting decreased from 96 to 67 days. However, the average number of days since the last all-staff meeting increased from 32 to 35 days.

For the health workforce category, we found increases in evidence of minimum required education for all staff roles; use of formal performance reviews; and use of staff duty roster. For the WASH category, we found increases in three out of the seven indicators—existence of a toilet to meet the needs of people with disabilities; separate toilets for patients and staff; and separate toilets for men and women. We found a decrease in the availability of hand hygiene materials both at toilets and points of care.

We found no changes in the indicators of service delivery, health information systems, and health financing.

### Interviews

In conducting an analytic induction of the semi-structured interviews, we found seven recurring themes: the importance of mentorship; leadership and governance; WASH; service delivery; community engagement; health equity; and self-efficacy.

#### The Importance of Mentorship

In interviews, participants reported that the mentorship component played a key role in their success. According to participants, mentors reinforced what they learned in class, explained concepts they were still confused about, and reminded them to complete their homework.

#### Leadership and Governance

In the area of leadership and governance, all health center chiefs reported using a more collaborative leadership approach after participation in the training. They now encouraged staff to share ideas about decisions that affected the whole health center. Health center chiefs reported involving staff members in tasks like technical assistance, budget planning, and annual planning. Health center chiefs also reported learning the importance of recognizing good performance and encouraging and motivating staff. Further, participants reported taking the initiative to solve issues at the health centers, rather than waiting for direction from the Operational District, Provincial Health District, or NIPH. For example, to address ambulance shortages in their rural district, the Koh Ke Health Center Chief and a staff member set up four donation boxes in the community, with proceeds paying for transport to the referral hospital, which is one and a half hours away. In addition, participants highlighted learning the importance of developing a better understanding of community needs, experiences, and access. For example, six health centers used lessons on mapping to create community maps allowing them to visualize the households in their catchment area; health center staff planned to use these maps to reach out to villagers that did not seek service at the health center.

Participants also reported learning the value of planning, particularly as it relates to implementing activities. Additionally, each health center involved in the training created four to six new standard operating procedures (SOPs), including clinical management guidance documents, with the exact number depending upon the needs of the health center. SOP topics included management of finances, outbreaks of food-borne illnesses, medical and non-medical waste, first aid for emergencies, first aid for newborns, road traffic-related accidents, and complications after childbirth. SOPs were a new concept for the health centers but were reportedly well-received and operationalized. At the time of our endpoint visit (January 2019), subordinate health center staff who did not participate in the IMPACT program were already trained or in the midst of receiving training on the SOPs. Staff at some health centers were already reporting success in their use. One example was at the Kamrieng Health Center, where staff used the new SOP for complications after childbirth. Following the SOP, they efficiently referred a woman to the hospital who had significant blood loss after giving birth.

#### Water, Sanitation, and Hygiene

In the area of WASH, we found that participants improved waste management at their health centers, increasing the number of waste bins from three to four or five, and ensuring separate bins for different waste types (e.g., non-medical and medical including pathological, toxic, infectious, and needles). Some health centers also created structures to store waste in the rainy season and burn it in the dry season. During our observational visits, we found a 10% decrease in the availability of hand hygiene materials and toilets at points of care (Table 5), however, in our interviews, staff at four health centers reported an increase in staff usage of hand hygiene materials at points of care and toilets.

#### Service Delivery

Although the service delivery indicators in the quantitative checklist did not change from baseline to endpoint observational visits, participants used knowledge from the course to improve the patient experience at their health center. Nine out of 10 health centers posted a board outside the health center directing patients where to go based on their needs.

Some health centers reported that the greatest change due to the training was instituting a patient triage system. For example, the Chrey Health Center now gives patients color coded squares upon arrival to define the severity of their condition and ensure those that need care urgently are prioritized.

#### Community Engagement

Participants reported learning new ways to communicate with the community and how to use the community as a resource in their work at the health centers. As described above, participants either planned to visit the community and reach out to community leaders or had already done so by the time of our visits. For example, at the Boribo Health Center, the chief and staff member realized during the training that they can begin mobilizing needed resources for the health center from the community rather than waiting to receive them through provincial health department channels. The health center is now being more resourceful by gathering wood from villagers and local non-governmental organizations to build a pre-delivery room for pregnant women.

#### Health Equity

Participants also reported health equity was a new concept for them and that it had an impact on the way they viewed their work. This new perspective led to changes in patient flow and setting up triage systems. At one health center, a chief reported that she's delegating tasks and responsibilities to her staff more equitably without regard to factors such as age or socioeconomic status.

#### Self-Efficacy

In the area of individual self-efficacy, participants reported feeling more confident to help the chief, share their ideas at staff meetings, and provide feedback to other staff on issues like tardiness. Participants also reported sharing the knowledge they learned at the training with other staff. This knowledge-sharing, in turn, encouraged other staff to share their knowledge with the rest of the team.

## Discussion

From these findings, we determined that the program did achieve its intended outcome—participants reported an increase in competence over seven key management areas. Through observational visits and interviews, we found that participants applied what they learned from the program. This finding is consistent with findings from the Philippines Field Management Training Program, a management capacity building program for local health workers similar to IMPACT. The Philippines Field Management Training Program found that “the results…provide compelling near-term evidence of how the skills from the training are being used by participants to identify, prioritize, and solve day-to-day managerial problems” (14).

These gains in management competence may not have been possible without mentorship support. Post-training support is a necessary aspect of effective training design (15). IMPACT mentors provided guidance and support while participants were at the health centers, bridging the gap between classroom training and experiential learning. One participant shared that it was easier to apply what they learned in the classroom with the mentors' support and guidance. For future health workforce capacity building programs, funders and program implementers should consider mentorship an essential aspect to ensure participant understanding and skill application.

During baseline observational visits, we saw that health facilities largely did well in the areas of service delivery, health information system, and health financing. However, endpoint interviews captured how the participants used the training to improve factors outside of the indicators in the quantitative checklist, particularly in the areas of service delivery, community engagement, and self-efficacy. These factors are demonstrative of more comprehensive management and leadership capability.

The WHO's key components of a well-functioning health system can provide a useful guide for funders and program implementers in how they build and tailor their health workforce capacity building programs, as well as how they measure the impact of these programs on the health system. We found the training positively contributed toward building four out of six of the WHO's building blocks for a well-functioning health system—leadership and governance, health information systems, human resources for health, and service delivery (Table 6) (8). We found no evidence that capacity improved around health financing and essential medical products and technologies.

**Table 6:** IMPACT Cambodia participants' contributions to related WHO key components of a well-functioning health system.

**WHO key component**	**IMPACT Cambodia participants' contributions to related WHO key component**
Leadership and governance	Participants' creation of plans
	Involving staff in creating plans
Health information systems	Identifying and analyzing geographic locations within catchment areas where residents who least utilized health services lived
	Developing targeted plans for community outreach to areas where residents who least utilized health services lived
Human resources for health	Conducting formal performance reviews
	Utilizing collaborative leadership approach
	Encouraging staff to share ideas about decisions related to health centre
	Recognizing positive performance
	Providing encouragement and motivation staff
Service delivery	Developing and utilizing SOPs
	Instituting patient triage systems
	Improving WASH practices at health centre
	Creating and displaying maps directing patients where to access health services
Health financing	No evidence
Essential medical products and technologies	No evidence

*WHO, World Health Organization; IMPACT, Improving Public Health Management for Action; SOPs, Standard Operating Procedures; WASH, Water, sanitation, and hygiene*.

For leadership and governance, participants' creation of plans, and more importantly, staff involvement in creating those plans, can help improve health outcomes in the complex, ever-changing environment of the health system (16). Yet, effective leadership and governance are only possible with accurate information on the health challenges and context in which the health system operates (8). To that end, by identifying and analyzing the geographical locations within their catchment areas where residents who utilized the least amount of health services lived, then developing targeted plans for community outreach aimed at encouraging those community members to visit the health center, participants will be able to improve access to care and the quality of services provided.

Leadership, governance and health information systems are cross-cutting components that provide the foundation for all the other building blocks, yet human resources for health is a key input in a well-functioning health system (17). A strong health workforce responds to the needs and expectations of the community, and is fair and efficient in achieving health outcomes with available resources (8). Through conducting formal performance reviews with staff, using a more collaborative leadership approach, encouraging staff to share ideas about decisions that affected the health center, recognizing positive performance and providing encouragement and motivation to staff, participants are creating enabling work environments for the health workforce, which encourage them to focus on the needs and expectations of the community.

Health systems can only be successful in producing health outcomes if they deliver the necessary services. Service delivery crucially depends on the development of standards and guidance to ensure access and quality of care (8). Through developing and utilizing SOPs, instituting patient triage systems, improving WASH practices at the health centers, and creating and displaying a map directing patients where to access health services, participants are ensuring access, greater satisfaction, and higher quality of care for their patients and the community.

While the training did not appear to have an impact on the two remaining building blocks of a health system (health financing and essential medical products and technologies), this could be due to decentralization in the Cambodian health system. Implementation of health programs is the responsibility of provincial health departments rather than the Ministry of Health. Due to decentralization, provincial health departments are the focal point for allocation of resources to operational health districts and health centers (18). Therefore, we believe expanding public health management training to health center staff nationwide, particularly to provincial health department managers, would be beneficial for Cambodia's health system.

One strength of this evaluation is the utilization of a mixed methods approach, which permitted a triangulation of results that discretely quantitative or qualitative research approaches alone would not have achieved. A single methodology would have been inadequate to examine the impact of the training program in a complex environment such as a health system (19). While evaluation findings are not generalizable (20, 21), the mixed method approach is replicable and may prove beneficial for other countries or funders to evaluate their health workforce capacity building programs. Yet we agree with Yeager and Bertrand that evaluations of health system strengthening programs, particularly ones focused on management and leadership, require a significant investment of resources, which may be a deterrent for some funders or program implementers (5).

This evaluation has several limitations. First, self-report data may lead to a misestimation of abilities when compared with objective assessments. This may be compounded by issues of comprehension and memory recall. To address this response bias, we used the observational visit checklist as an objective assessment at baseline and endpoint to compare with the interview data. Second, these evaluation results are not generalizable outside of this program in Cambodia, but do demonstrate the short-term results of the implementation of such a program (20, 21). Third, since we used human translation to translate collected data from Khmer to English for analysis, it is possible that context was lost in translation. Fourth, we did not validate participants' interview responses with independent sources, so it's possible our own personal experiences may have resulted in methodological bias. To address this bias, we've attempted to clearly and accurately present participants' experiences in all cases (22). Lastly, the evaluation was constrained by time and our inability to assess the long-term impact of the training on graduates and the health outcomes in their communities. A long-term study that compares health centers with IMPACT graduates to those without IMPACT graduates and utilizes data from Cambodia's Health Equity and Quality Improvement Program could potentially measure the cascading effect of training participation on health outcomes.

The evaluation findings may have several practical implications for funders and program implementers, particularly those engaged in building and tailoring health workforce capacity building programs. In terms of program design, purposeful mentorship may be instrumental to ensuring participant understanding and skill application. In addition, trainings focused on competency development for frontline health managers and providers can be highly effective in skill-building and behavior change in health facility operations. Further, focused training of this kind may lead to demonstrations of enhanced leadership, self-efficacy, cooperation amongst staff, as well as improved service delivery and community engagement.

The evaluation approach may also provide lessons for global health capacity-building programs. Rigorous evaluations of such programs do require a significant investment of resources. However, this investment is critically important to understand the possible effects of these programs. Moreover, they are important to building the evidence-base around workforce capacity development efforts, particularly in the areas of health system management and leadership where there has been limited study.

We hope this evaluation encourages further research into the impact of skilled public health management on health systems, not only in low- and middle-income countries, but also in well-resourced nations. Although the Global Health Security Agenda identifies improved public health management capacity as essential for achieving its goals (23), many health systems still do not invest in or prioritize developing public health management capacity (24). The COVID-19 pandemic has elevated the urgency of this work, particularly in the managerial areas of coordination, community engagement and trust, planning, and adaptive leadership (25). Thus, continued investment and evaluation of workforce development efforts of this kind may be critical to addressing underlying health system issues and improving public health outcomes.

## Data Availability Statement

The de-identified data that supports the findings of the study are available on request from the corresponding author.

## Ethics Statement

The studies involving human participants were reviewed and approved by U.S. Centers for Disease Control and Prevention Center for Global Health's Institutional Review Board and the Cambodia National Ethics Committee for Health Research. The patients/participants provided their written informed consent to participate in this study. Written informed consent was obtained from the individual(s) for the publication of any potentially identifiable images or data included in this article.

## Author Contributions

KH, PW, PI, CC, MK, VL, and EW equally involved in the conceptualization and design of the program. CH led the data collection, analysis, and interpretation. CH, KH, PW, and CC drafted the manuscript. CH, PI, MK, VL, and EW provided critical revisions to the manuscript. All authors were involved in designing the evaluation of the program and approved the final version of the manuscript.

## Funding

This publication was supported by Grant or Cooperative Agreement numbes 5UGH001873 and 1NU2HGH000044-01-00, funded by the U.S. Centers for Disease Control and Prevention. This project was supported by grant numbers 5NU2GGH000990-05-00 and 6NU2GGH002124-01-01, funded by the Korea International Cooperation Agency. This project was supported in part by an appointment to the Research Participation Program at the U.S. Centers for Disease Control and Prevention administered by the Oak Ridge Institute for Science and Education through an interagency agreement between the U.S. Department of Energy and the U.S. Centers for Disease Control and Prevention.

## Author Disclaimer

The views expressed in the submitted article are solely the responsibility of the authors and do not necessarily represent the official views of the U.S. Centers for Disease Control and Prevention, the U.S. Department of Health and Human Services, the Korea International Cooperation Agency, the Task Force for Global Health, Inc., or Training Programs in Epidemiology and Public Health Interventions Network.

## Conflict of Interest

The authors declare that the research was conducted in the absence of any commercial or financial relationships that could be construed as a potential conflict of interest.

## Publisher's Note

All claims expressed in this article are solely those of the authors and do not necessarily represent those of their affiliated organizations, or those of the publisher, the editors and the reviewers. Any product that may be evaluated in this article, or claim that may be made by its manufacturer, is not guaranteed or endorsed by the publisher.
